# Characterization of Filigree Additively Manufactured NiTi Structures Using Micro Tomography and Micromechanical Testing for Metamaterial Material Models

**DOI:** 10.3390/ma16020676

**Published:** 2023-01-10

**Authors:** Thomas Straub, Jonas Fell, Simon Zabler, Tobias Gustmann, Hannes Korn, Sarah C. L. Fischer

**Affiliations:** 1 Fraunhofer Cluster of Excellence Programmable Materials, 79108 Freiburg im Breisgau, Germany; 2 Fraunhofer Institute for Mechanics of Materials IWM, 79108 Freiburg im Breisgau, Germany; 3 Lightweight Systems, Saarland University, 66123 Saarbrucken, Germany; 4 Fraunhofer Institute for Integrated Circuits IIS, 91058 Erlangen, Germany; 5 Fraunhofer Institute for Machine Tools and Forming Technology IWU, 01187 Dresden, Germany; 6 Fraunhofer Institute for Nondestructive Testing IZFP, 66123 Saarbrucken, Germany

**Keywords:** NiTi, shape memory alloys, lattice structures, filigree structures, additive manufacturing, micro tomography, micromechanical testing, metamaterials

## Abstract

This study focuses on the influence of additive manufacturing process strategies on the specimen geometry, porosity, microstructure and mechanical properties as well as their impacts on the design of metamaterials. Filigree additively manufactured NiTi specimens with diameters between 180 and 350 µm and a nominal composition of Ni_50.9_Ti_49.1_ (at %) were processed by laser powder bed fusion in a first step. Secondly, they structures were characterized by optical and electron microscopy as well as micro tomography to investigate the interrelations between the process parameters, specimen diameters and microstructure. Each specimen was finally tested in a micro tensile machine to acquire the mechanical performance. The process strategy had, besides the resulting specimen diameter, an impact on the microstructure (grain size) without negatively influencing its quality (porosity). All specimens revealed a superelastic response while the critical martensitic phase transition stress decreased with the applied vector length. As a conclusion, and since the design of programmable metamaterials relies on the accuracy of FEM simulations, precise and resource-efficient testing of filigree and complex structures remains an important part of creating a new type of metamaterials with locally adjusted material behavior.

## 1. Introduction

Mechanical metamaterials promise to revolutionize the way we view materials and tune materials beyond their bulk properties. Metamaterials consist of geometrically designed unit cells that form the basis of the material behavior [1,2]. The parameters of the unit cell can be tailored globally to achieve homogeneous unconventional properties (e.g., auxetic behavior with negative Poisson’s ratio) or locally to achieve materials with even more complex behavior such as adaptive shape, programmable damping properties or multiple stable states [3,4,5,6]. One of the main parameters used to tune the properties of such materials are varying feature sizes (e.g., strut diameter or shape) of filigree, lattice-like elements [7,8,9].

Due to their complexity, the design of metamaterials is usually performed based on simulations first. Accurate material models are required in order to increase the fidelity of the simulations [3,10]. As the mechanisms are often based on high strains, the use of linear elastic models yields wrong property predictions, and it is necessary to utilize more complex material models. Moreover, in the case of filigree structures, it is important to understand size effects, because dimensions of unit cell parameters are used to tailor the unit cell behavior (e.g., mechanical performance). While many studies exist looking at mechanical properties based on macroscopic bulk [11,12] specimens in the range of several millimeters, the mechanical performance and cyclic performance of filigree structures, such as vertical struts (as smallest subsets of lattice structures), have not been studied in great detail. This is, however, of great importance for the manufacturing and application of metamaterials [13,14].

Metallic metamaterials offer a unique combination of promising properties, combining mechanics beyond bulk properties with higher tolerance for environmental conditions such as heat or moisture when compared to most polymeric materials [15]. Even though structural modifications can lead to metallic metamaterials with high elongation, their natural capability for elastic deformation is rather small with respect to, for instance, polymers, which limits their widespread application. Therefore, alloys with superelastic properties at room temperature such as shape memory alloys (e.g., Ni-rich NiTi) are intensively studied to manufacture mechanical metamaterials which can withstand high local strains in unit cells [16]. To be able to use these alloys as metamaterials, conventional manufacturing methods such as investment casting and machining can only be used in a limited way. Thus, progress needs to be made in additive manufacturing with superelastic alloys as it offers high design flexibility [17]. Additive manufacturing of intricate structures, e.g., via Laser Powder Bed Fusion (LPBF), has high requirements concerning manufacturing defects [18] and is subject to a complex interrelation of the applied process parameters with the corresponding microstructure and material properties.

In the present work, we studied the mechanical properties of cylindrical NiTi struts as a possible subset of a metallic metamaterial produced via LPBF with diameters in the range of 180 µm to 350 µm. This micro scale is not sufficiently addressed in the literature so far as studies focus on macroscopic samples with dimensions in the mm to cm range. Findings on non-destructive characterization as well as on micromechanical testing to analyze the effect of feature size and scanning strategy on the quasi-static properties of the struts are reported. Finally, the impact of the size effects using a simulation of a simplified metamaterial model was visualized to showcase the importance of including size effects in material models. Accurate elastic–plastic material models as presented here are necessary for the design of complex metamaterials, and more characterization effort across the metamaterial design is necessary in the scientific community.

## 2. Materials and Methods

In the following section, experimental and computational methods used for this research work are explained.

### 2.1. Additive Manufacturing of NiTi Specimens

**Figure 1:**
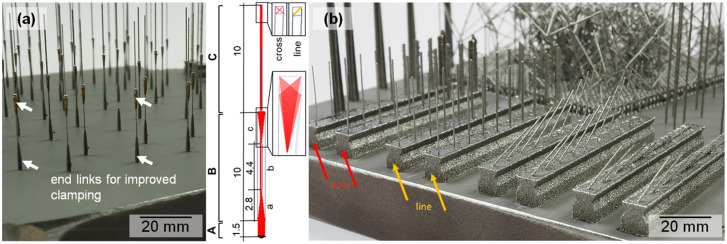
Macroscopic images of the (**a**) filigree tensile specimens (20 mm in height) used for mechanical testing in a self-assembled micro testing setup (see Figure 2). The tensile specimens span from handle to handle while the upper part was clipped from the specimen and kept for further analysis (for further explanations and the schematic drawing please see the text in Section 2.1). An image of single struts with varying diameters (*P* = 200 W, *v* = 500 mm/s) is shown in (**b**). The arrows mark the specimen rows produced with line (yellow) and cross (red) quasi-P scanning strategy. The highlighted specimens, with respect to their shape and diameter, are compared in Figure 3.

Filigree Ni_50.9_Ti_49.1_ (at %) specimens (single struts, micro tensile specimens; see Figure 1) were manufactured at Fraunhofer IWU by LPBF using a M2 Cusing system (Concept Laser GmbH, Lichtenfels/Germany). For the processing of the aforementioned filigree specimens (strut diameter: >150 µm) under argon atmosphere, a layer thickness of 25 µm and a relative fine powder (PSD: *d*_10_ = 13 µm, *d*_50_ = 21 µm, *d*_90_ = 35 µm) were used.

The specimens shown in this study were produced on NiTi substrate plates using an adapted hatching workflow (quasi-P scanning strategy [19]). The approach regarding processing (scanning strategy and process parameter optimization) is reported elsewhere [20]. The specimen design consists of three sections (see Figure 1a). Section A is a machining allowance, which was removed before the tensile tests. Section B was used as a tensile test specimen and section C as a filigree strut and reference specimen for further investigations (e.g., microstructural analysis). Section B was designed in such a way that it consisted of the end links (see Figure 1a) a and c, and the tensile testing subsection b. Section C was manufactured using the same parameters as subsection b in section B and was removed before tensile testing.

The diameter of the filigree specimens (single struts) was varied by using short straight scanning lines. Three different settings regarding the vector length factor were used: (i) short exposure (smallest strut diameter, designated as “1”), (ii) medium exposure (small strut diameter, designated as “5”) and (iii) long exposure (increased strut diameter, designated as “10”). The vector length factor multiplied by √2 × 10^−2^ gives the vector length in mm. The described scanning settings regarding the line length were applied to two scanning types: line and cross quasi-P scanning strategy. In other words, the filigree specimens produced were tailored (shape, diameter) by solely adjusting the aforementioned scanning settings. The applied process parameter, laser power (*P*), scanning speed (*v*) and laser focus diameter were held constant at 200 W, 500 mm/s and 100 µm, respectively. In order to improve the clamping during tensile testing, single struts were produced in such a manner that the end links were, irrespective of the applied exposure for section B (see sketch in Figure 1a), additionally hatched with a cross-scanning (*P* = 100 W, *v* = 500 mm/s, long exposure “10”).

**Figure 3:**
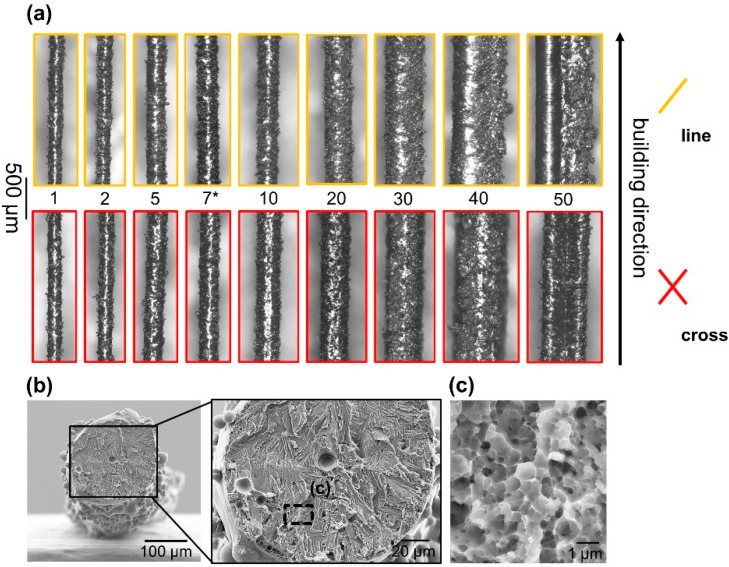
Overview of single struts fabricated with different scan vector lengths (**a**). The color-code corresponds to the highlighted specimens in Figure 1b, viz. line and cross quasi-P scanning. Please note that the indicated exposures are only shown schematically perpendicular to the building direction. As marked (*), a selected specimen from previous tests [20] was used for a detailed analysis of the fracture surface after tensile loading (**b**). A magnified image of the fracture surface is shown in (**c**). For tensile testing, specimens produced with vector length factors from 1 (“short” scanning vectors) to 10 (“long” scanning vectors) were chosen.

### 2.2. Microscopical Analysis

For the analysis of the strut diameter and the investigation of the corresponding shape, mainly 2D images of selected specimens were taken using an optical microscope Eclipse ME600 (Nikon, Tokyo, Japan). In order to improve the specimen handling, single struts were produced as a whole specimen setup (see Figure 1b). A total of 5 measurements along the struts were considered for obtaining a mean value of the strut diameter.

In addition, selected specimens and their corresponding surfaces (after fracture) were investigated using a scanning electron microscope (SEM) Supra-25 (Zeiss, Oberkochen, Germany). Images were recorded using an SE (surface appearance) and BSE (chemical homogeneity) contrast detector. EBSD scans of selected specimens were performed with a step size of 0.25 µm. EBSD analysis was used to investigate the microstructure with respect to the grain size. For EBSD analysis, the OIM Analysis^TM^ 8.6 software (by EDAX, Mahwah, NY, USA) was used with a grain detection angle of 15° and a minimum of 10 pixels per grain. The step size of EBSD scans was 0.25 µm and the grid was hexagonal.

### 2.3. X-ray Micro-Computed Tomography (µCT)

The µCT measurements were performed with the MetRIC scanner (Fraunhofer MRB Würzburg—Germany) using a voltage of 140 kV and 8 W power on two different length scales. A total of 42 specimens were scanned with a voxel size of 6.2 µm and a total amount of 1800 projections in order to cover the entire length of the tensile specimen and to evaluate dimension and geometry of the struts. To better resolve pores, the specimens were further scanned with a voxel size of 2.6 µm, acquiring 2160 projections. Volume reconstructions were performed with the software pyXIT-LRM (version 2020).

The µCT data were segmented via gray value thresholding and visualized by the software Avizo (version 9.0). Quantitative analyses of pores and matrix were conducted directly through Avizo.

### 2.4. Micromechanical Testing

**Figure 2:**
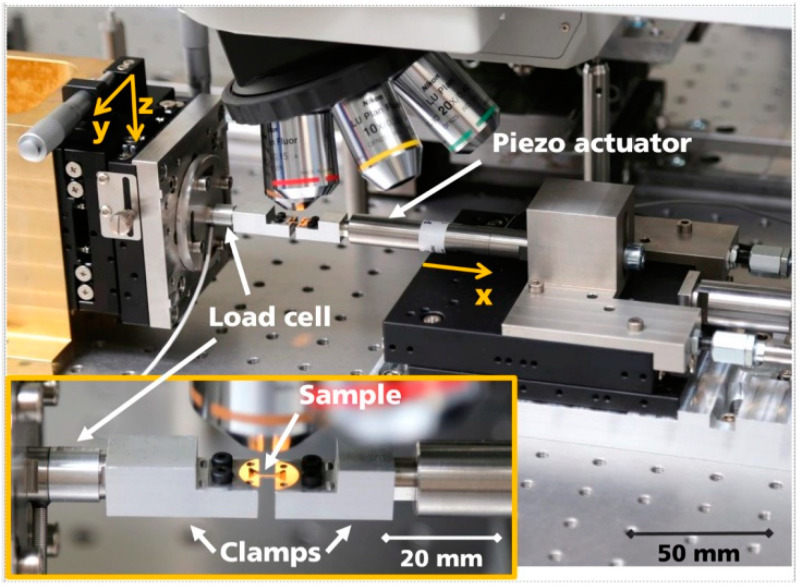
Images of the custom-built micromechanical testing device used in the presented work.

In order to be able to characterize the material behavior of NiTi micro specimens, a custom-built micromechanical testing device (Figure 2) was used. It is based on the work of Kennerknecht et al. [21] and Straub et al. [22] and has been further developed over the years by Fraunhofer IWM.

Quasi-static tensile tests were performed in order to assess the overall mechanical properties. The specimens were pulled apart by the actuators at a constant velocity of 1.25 µm/s. The force applied was measured with a 200 N load cell and recorded at a constant time stamp. Images of the specimen surface were taken continuously by the camera at the same exact time. The frequency of the force and image acquisition was directed according to the specified elongation rate. To obtain the typical stress–strain curve for quasi-static tensile tests, the strain of the specimen was calculated from the recorded images. The programming environment MATLAB and the image correlation code developed by Senn et al. [23] were used. For the image correlation, a virtual grid was applied to the first image of the series. Each image was then compared to the initial one. In this way, the engineering strain can be determined for each picture in the series. Since the exact time of recording was stored with the images, the calculated engineering strain can be assigned to a force measurement value via the time stamp. The measured force values were converted into an engineering stress by means of the measured specimen cross-section. The stress–strain value pairs calculated in this way could then be plotted in a stress–strain diagram.

### 2.5. Numerical Simulations

Two-dimensional unit cell models were designed with SolidWorks 2020 (Dassault Systèmes, Paris, France). Numerical simulations were performed based on those models with Comsol Multiphysics 5.6. (COMSOL AB, Stockholm, Sweden) using the solid mechanics module and stationary study. Custom material models were generated with results from micromechanical testing based on an elastoplastic formulation.

## 3. Results and Discussion

The main focus of the present study was to investigate the interplay between the diameter of additively manufactured struts and their corresponding properties (geometrical and structural imperfections, superelastic behavior under tensile loading) considering two different scanning strategies to inform future material models for metamaterial design. In a first step, struts with a diameter range between 180 µm and 350 µm were manufactured by adjusting the scanning strategy.

### 3.1. Influence of Scanning Strategy on Strut Diameter and Morphology

To obtain lattice structures or single struts with little residual pores, it is important to design the process parameters in such a way that sufficient melting without overheating the melt pool is present. Due to the detailed investigations on NiTi lattice struts [20], it has been found that a line energy input of 0.4 J/mm (*P* = 200 W, *v* = 500 mm/s) is a suitable value for the fabrication of single struts, both in vertical as well as tilted orientation (see Figure 1b). It is noteworthy that filigree structures with the aforementioned parameters (see Section 2.1) can be produced with diameters below 200 µm using a conventional LPBF machine. This is surprising regarding the laser beam diameter and other findings [24,25]. However, a controlled exposure of the applied material via quasi-P scanning [26] results in a very short interaction time of the laser with the local powder bed, which is the explanation for the aforementioned effect. In other words, to fabricate filigree structures in a range of 100 to 200 µm, the use of adapted approaches such as micro selective laser melting [27] is, therefore, not needed.

As summarized in Table 1, the strut diameter can be varied systematically by increasing the scan vector length. This has not been observed so far by keeping the vector length constant and, in an analogous way, adjusting the line energy input. The smallest vector length factor applied (“1”) leads to a strut diameter of approx. 180 µm, irrespective of the scanning type (see line and cross in Table 1). This lingers until the vector length factor reaches values above 2, where cross-like scanned struts are always slightly enhanced in terms of their diameter (see also Figure 3a). This, however, has only been observed for applied vector length factors of 2 to 20. If longer vectors are used, line-like scanned struts are enhanced in diameter in comparison to their cross-scanned counterparts. This can be attributed to the fact that double scanning for cross-exposure occurs in a relatively small area of a few hundred micrometers, which causes the laser to jump in and out differently in contrast to a single exposure (line). This has not been observed yet but is also of limited importance as the main goal of our approach is to stay in a process window where cylindrical struts with a diameter of around 100 to 300 µm (vector length factors with a value smaller than 20, see Figure 3a) can be produced.

Not only does the diameter shift towards higher values, but also the strut morphology significantly changes when increased vector length factors (VLF) are used (VLF > 30). Typically, the as-built struts are relatively rough due to small residual particles that were not melted but attached to the solidified surfaces (see also Figure 3b). As the vector length factor increases, a spherical shape turns into a rather ragged morphology (see examples for exposure 40 to 50 in Figure 3a) which follows the nature of the applied scanning (line: fin-like, cross: cross-like). Hence, these specimens are not suitable for producing homogeneous and well-defined parts later and were, firstly, not investigated in detail in our study and, secondly, not considered for tensile test specimen fabrication. Adequate mechanical performance of single struts can only be guaranteed if they, besides other factors such as microstructure and surface quality, obtain a minimal pore volume [7]. For instance, the image of a fractured, line-scanned strut (approx. 235 µm, see also exposure “7*” in Figure 3b) confirms these findings. The strut reveals signs of ductile fracture but failed in an atypical early stage for NiTi [28], at around 1.8% strain, as shown in detail in a former publication [20].

To understand the influence of an increasing VLF and the type of scanning strategy on the microstructure and mechanical behavior under tensile stress, geometrically optimized single struts were manufactured with a vector length/exposure of 1, 5 and 10. Their morphology and quality (density, pores size, etc.) were analyzed in advance via µCT as described in the following section.

### 3.2. 3D Strut Characteristics

**Figure 4:**
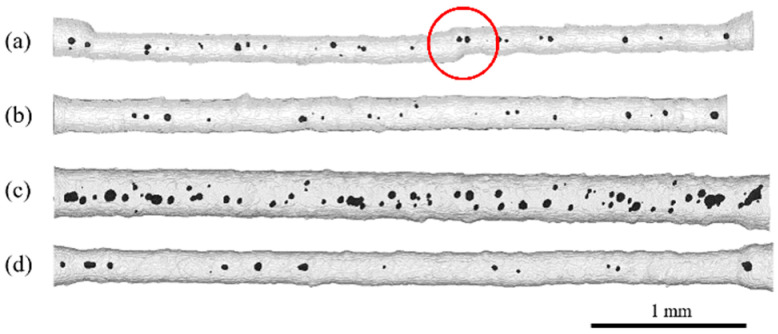
Reconstructed 3D images of µCT scans of (**a**) specimen L1-D with offset defect (red circle), (**b**) specimen C1-B, (**c**) specimen L10-F and (**d**) specimen C1-C showing segmented struts (transparent view) with pores in black.

**Figure 5:**
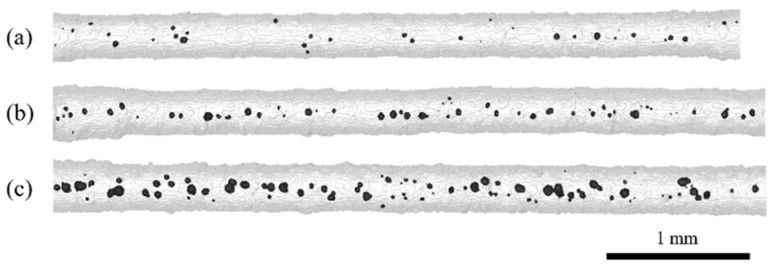
Reconstructed 3D images of µCT scans of (**a**) specimen C5-A, (**b**) specimen C5-G and (**c**) specimen C5-C showing segmented struts with pores in black. Struts within the same type of scanning strategy show high reproducibility in terms of dimensional accuracy but also variations in pore volume ((**a**) = 0.15%, (**b**) = 0.37%, (**c**) = 1.28%).

**Figure 6:**
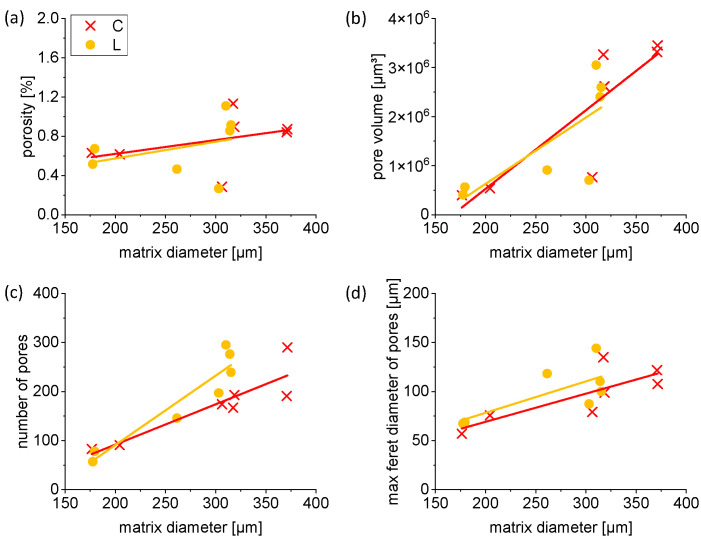
Porosity, pore volume, number of pores and maximum Feret diameter depending on matrix diameter of struts for scanning types L and C. Graph (**a**) shows an overall small porosity, slightly increasing with rising matrix diameter. Pore volume (**b**), number of pores (**c**) and maximum Feret diameter of pores (**d**) increase with rising matrix diameter. The lines are intended to guide the eye of the reader and do not represent linear fits.

Following the optical analysis of the struts, X-ray micro-computed tomography was performed to evaluate the morphology of the filigree struts (dimension, geometry, surface roughness) according to the measurements of the entire strut displayed in Figure 4 and Figure 5. The analysis of the internal structure (section B–subsection b, see Figure 1) was conducted according to the measurements with 2.7 µm voxel sampling to quantify pores and matrix parameters, as presented in Figure 6.

As shown in Figure 4 and Figure 5, the segmented struts are visualized with a transparent gray matrix volume and black pores. The entire struts and parts of the volume used for clamping are depicted at the sides. In general, all specimens indicate a relatively high dimensional accuracy; however, scanning type C shows an even more precise accuracy than scanning type L. It rarely occurs that struts show an offset defect, as displayed in Figure 4a, which might have been caused by a temporal shift in the already processed structure via local recoater blade contact. The evaluation of the strut surface shows an overall strong variation in roughness caused by partially melted particles sticking to the surface. The effect of different vector length factors can be clearly seen in the resulting strut diameter of specimen L10-F in Figure 4, which is significantly higher than the diameter of the other specimens with a vector length factor “1”. Regarding all measured specimens, the mean strut diameter is 198 ± 19 µm for vector length factor “1”, 282 ± 28 µm for vector length factor “5” and 344 ± 33 µm for vector length factor “10”. These values are precise representatives of the analyzed volumes and relatively close to the results gathered via optical microscopy (OM, cf., Table 1). Hence, for a higher number of specimens and high-throughput approaches (e.g., process development for various NiTi shape memory alloys), an OM analysis would be a promising way for a rather fast investigation of specimen arrangements, as highlighted in Figure 1b.

There is an influence of the two scanning types revealing a 15% higher strut diameter for scanning type C than for scanning type L regarding to each VLF. During C-type scanning, geometries are scanned in a cross-like manner. Thus, all structures are subjected to double scanning, which can explain the enhanced strut diameters in contrast to the L-type specimens (single-scan exposure).

Figure 5 shows three C5 specimens with the same scanning type and vector length factor. The high dimensional accuracy indicates a high reproducibility. However, the pore volume shows a rather strong variation within the group of the C5 specimens, varying between 0.15% and 1.28% porosity. The reason for this can be correlated to the applied fine powder feedstock (see [20], agglomerates and limited flowability) and thus to a varying local powder bed density which often directly reflects in part density and accuracy [29]. In other words, even with a non-ideal powder batch, promising results for the processing of filigree NiTi specimens have been obtained. The aforementioned residual porosity can be described as a rather low overall porosity with respect to other studies [8,18]. The average porosity of all 42 investigated specimens is 0.5% ± 0.28%. A minimum porosity of 0.8% and maximum porosity of 1.28%, as already stated, were noted.

To correlate the geometrical parameters revealed by µCT (voxel sampling of 2.7 µm) with the mechanical measurement data, the focus has been put on a set of specimens, which were investigated more precisely. Scanning types C and L show an overall similar behavior in terms of quantitative geometrical analysis. As mentioned before, the porosity of the struts is relatively low, which is also presented by the upper left graph in Figure 6, showing a porosity between 0.2% and 1.2%. It is noteworthy that the residual porosity is only slightly increasing with the matrix diameter of the struts. A wide manipulation of possible geometries (orientation, combination of various locally adapted struts) is, therefore, possible. In other words, the selected scanning type and vector length factors for manufacturing filigree LPBF struts are suitable to obtain specimens with a low porosity and high reproducibility in terms of dimensional accuracy.

The revealed pores in the struts show a slight increasement of their volume, amount and maximum Feret diameter with rising matrix diameter (strut diameter) for scanning types C and L. Due to the constant mean pore diameter of 19 µm ± 3 µm, independent of the matrix diameter of the struts, the rising number of pores results in an increment in the pore volume. However, the maximum Feret diameter of pores increases with rising matrix diameter. Thus, the influence on the mechanical properties must be precisely characterized (see Section 3.3).

### 3.3. Microstructure and Micromechanical Behavior

**Figure 7:**
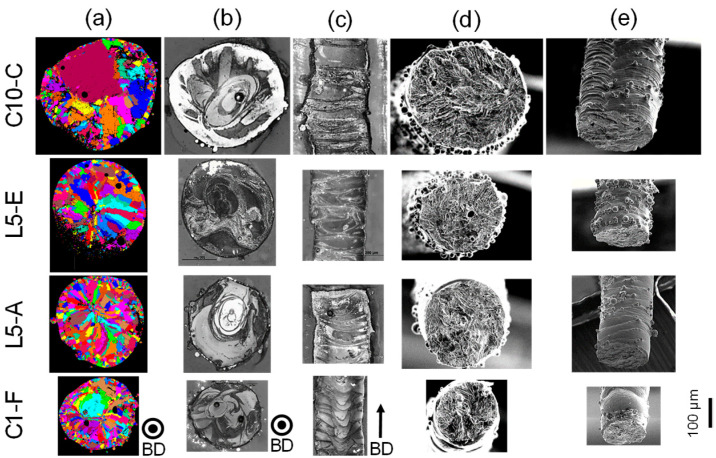
Overview of selected strut specimens and their corresponding microstructural (SEM images; building direction—BD—is highlighted; (**a**) EBSD grain map, (**b**) etched perpendicular section and (**c**) etched longitudinal section) and fracture surface fingerprints (SEM images; (**d**) fracture surface and (**e**) tilted fracture surface).

In order to correlate the influence of the scanning strategy on the resulting material properties, microstructural analysis was carried out first. The conducted EBSD scans, shown in Figure 7a, imply that relatively fine grains are formed in the struts, which is usually the case for LPBF-processed specimens across different part-length scales (high local cooling rates) [30]. In the case of the applied scanning strategy, the interaction time per layer in respective areas is very short, which is also a key issue for the small adjustable diameters presented in this work (see previous sections).

The grain distribution and sizes are changing to some extent depending on the applied scanning type and vector length (cf., Figure 7a). In other words, they follow our findings from the geometrical analysis, viz., an increased vector length corresponds to an enhanced strut diameter. As it can be seen in Table 2, the average grain size increases with the vector length. Furthermore, the specimens fabricated with a cross-type scanning strategy (see C1-F and C10-C in Figure 7) obtained a significant difference in the grain morphology and distribution compared to the line-type counterparts. Besides the small and equiaxed grains in the surface near areas, the grains that were formed in the strut center show a similar morphology and do not follow the heat flow. Thus, columnar grains as seen for L specimens were not observed. The main reason for this is the applied double-exposure for cross-type scanning. All these specimens were scanned with two crossing scan vectors (longitudinal and transverse direction) in each layer in a step-by-step-like manner. An illustration of this is presented in Figure 7b, where the microstructure in the scanning direction is highlighted (in build direction: Figure 7c). Hence, every layer is locally and immediately remelted after the first scan. This causes a rather slow (heat accumulation due to double scanning) and more homogeneous solidification, which can explain the grain size and morphology differences. To further understand the grain formation with respect to the applied scanning strategy is part of a future work and beyond the scope of the present study.

Subsequently, micromechanical experiments were conducted on the L- and C-type specimens. The tests were carried out following the testing methodology described in Section 2.4. The fracture surfaces are depicted in Figure 7d as well as in Figure 7e and revealed no specific differences with respect to the applied scanning strategy or strut diameter.

The recorded stress–strain curves of the as-built struts showed typical mechanical behavior known for Ni-rich NiTi shape memory alloys (austenitic at room temperature) [1]. Firstly, a linear elastic regime occurs during loading. It is known that the austenitic phase (B2) is stable under these conditions irrespective of the applied stress. After crossing a critical phase transition stress, the curves, secondly, reached a pronounced plateau. This is known as pseudo- or superelasticity and is triggered by the stress-induced martensitic transformation (B19’) [31]. Finally, the slopes of the curves increase again (elastic regime of detwinned martensite), followed, thirdly, by a plastic deformation of the aforementioned detwinned phase and a failure of the specimen.

**Figure 8:**
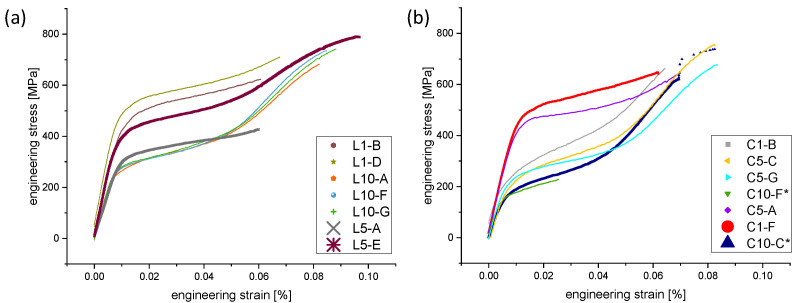
Engineering stress–strain curves from quasi-static tests of L- (**a**) and C-type (**b**) specimens. The microstructure and fracture surface of the highlighted specimens (curves in bold) were investigated in detail (see Figure 7). The experimental tests for the specimens marked with * were manually stopped before failure.

**Figure 9:**
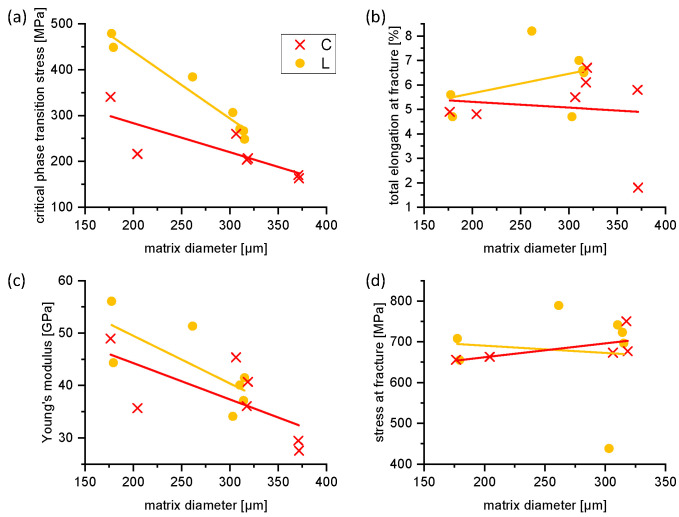
Critical phase transition stress (**a**), total elongation at failure (**b**), Young’s modulus (**c**) and stress at failure (**d**) plotted as a function of matrix diameter (strut diameter). The lines are intended to guide the eye of the reader and do not represent linear fits.

It can be observed in Figure 8 that the L- and C-type specimens have a large scatter on the critical phase transition stress, the Young’s modulus as well as the achieved elongation. This is directly related to the specimen diameter (and with it to the grain size), as can be seen in Figure 9. The “thicker” the specimens, the lower the critical phase transition and the Young’s modulus. In addition, knowing that the total elongation to failure mainly depends on the specimen quality (surface roughness, porosity), we are convinced that the scatter for those strain measurements has to be individually discussed in specific cases. The surface roughness is often a good performance indicator of a mechanical component, since irregularities on the surface may form nucleation sites for cracks. This is nevertheless rather relevant for fatigue testing under cyclic loading than for quasistatic loading. A further process optimization or the use of a high-quality powder batch (e.g., using ultrasonic-atomized powders [32,33] could be therefore interesting aspects for future approaches and to improve deformability.

It is noteworthy that the different stress–strain curves (cf., Figure 8) can be related to the different microstructures of the manufactured struts. In other words, the critical phase transition stresses (cf., Table 2) show a strong trend. The specimen with the highest strut diameter (C10-C), for instance, presents the lowest critical phase transition stress (163 MPa). On the contrary, specimens with a minimal strut diameter and smaller grain sizes reveal higher critical phase transition stresses. This can be explained by the Hall–Petch effect. The Hall–Petch relation [34,35] defines that the yield strength in materials is in direct correlation to the grain size (“the smaller, the stronger”). As it was demonstrated that this general finding also holds for various shape memory alloys (start of pseudoplastic or pseudoelastic regime, stress at failure [36,37,38,39]), it can also serve as an explanation for the obtained interrelations and results.

### 3.4. A Metamaterial Model and Validation Perspective

In the following paragraph, the experimental findings are visualized with a simplified metamaterial unit cell inspired by previous research works [3]. Wenz et al. and other researchers used the strut dimension as a parameter to manipulate/program their unit cells with a specific behavior such as tailored, time-dependent Poisson’s ratio. The unit cells with a strut diameter of 180 µm and different material models are subject to gradually increasing stresses. Elastoplastic material models were set up to match the experimental data of this paper for two cases: structure dimensions of d ≈ 180 µm (E1 = 76 GPa; critical phase transition stress = 450 MPa) and d ≈ 310 µm (E1 = 40 GPa; critical phase transition stress = 270 MPa). The density was set to 6450 kg/m³ and the Poisson ratio to 0.3.

**Figure 10:**
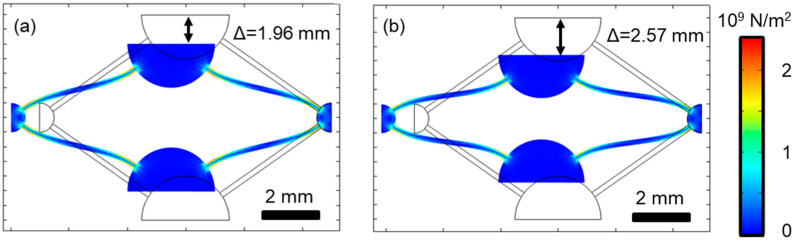
Deformation and resulting van Mises stresses of two unit cells with different material models subject to an identical stress (see text for more details). Material models are based on experimentally determined parameters for (**a**) d ≈ 180 µm and (**b**) d ≈ 310 µm, i.e., close to bulk properties.

The deformation and resulting van Mises stresses of two unit cells with different material models, subjected to identical stresses, are shown in Figure 10. Figure 10a illustrates the unit cell with matching dimensions and material model, while the unit cell highlighted in Figure 10b was assigned to a material model that has been experimentally found for larger structure dimensions and close to bulk properties of additively manufactured specimens. A stress of 50 MPa was applied to one side of the structure while the opposing side has a floating bearing boundary condition. A lateral contraction of 1.96 mm (i.e., 28%) was observed for the unit cell with the scaled material model and 2.57 mm (i.e., 37%) for the model with material properties not adapted to the strut diameter.

Assuming bulk properties for thin struts will result in large errors of the predicted lateral deformations on a single unit cell level, it will then translate to the system level. Therefore, the intention of this material model was to implement elastoplasticity and include size effects. Although two distinct material models were compared, it is necessary in the future to extend this approach to assign material model parameters as a function of structure dimensions automatically.

## 4. Conclusions

Filigree additively manufactured NiTi specimens with diameters between 180 and 350 µm with a nominal composition of Ni_50.9_Ti_49.1_ (at %) were processed by laser powder bed fusion. The diameter of the filigree specimens was varied by using different scanning strategies and different settings regarding the applied vector length factor.

It can be concluded from Section 3.2 that the specimens have a low porosity in general. The pore size and Feret diameter vary slightly with the strut diameter, but there is almost no variation in the overall porosity. The influence of the pores and surface topography (such as surface roughness) on the total elongation at failure and on the stress at failure was found to be small. This may not apply to cyclic tests, though, and is part of a future study.

Section 3.3 highlighted that the struts have a varying microstructure, especially different grain sizes, depending on the applied scanning strategy. This correlated well with the visible trend in the mechanical properties, such as the critical phase transition stress and Young’s modulus. The specimen with the highest strut diameter, for instance, presents the lowest critical phase transition stress. On the contrary, specimens with a minimal strut diameter and smaller grain sizes reveal higher critical phase transition stresses.

When scaling and manufacturing the unit cells, material models based on bulk properties and material models without adaptive parameters according to feature size will not lead to the simulation of realistic mechanical properties. Based on the simulations (Section 3.4) and the results from this paper, it becomes obvious that the design of programmable metamaterials with advanced properties rely on the interplay of a multitude of disciplines coming from both the manufacturing and simulation side. Therefore, precise and resource-efficient testing of filigree and complex structures remains an important part in connecting both worlds to create a new type of metallic parts with locally adjusted material behavior.

## Figures and Tables

**Table 1:** Summary of the filigree specimens produced for µCT analysis (Section 3.2) and micromechanical testing (Section 3.3). The nomenclature is related to the type of scanning (sequence, line length). The corresponding strut diameters measured by optical microscopy are also listed.

Specimen	Type of Quasi-P Scanning	Vector Length Factor	Strut Diameter (µm)	Morphology
L1	L-line	1	180 ± 9	Even and cylindrical
L5	L-line	5	221 ± 8	
L10	L-line	10	273 ± 6	
C1	C-cross	1	180 ± 6	
C5	C-cross	5	248 ± 7	
C10	C-cross	10	301 ± 7	

**Table 2:** Average grain size and critical phase transition stress of selected analyzed struts produced via C-type and L-type scanning strategies.

Specimen	Average Grain Size (µm)	Critical Phase Transition Stress (MPa)
C1-F	23	340
L5-A	18	306
L5-E	25	384
C10-C	78	163

## Data Availability

The data that support the findings of this study are available from the corresponding authors, T.S. and S.C.L.F., upon reasonable request.
